# Geographical prevalence of SARS-CoV-2 variants, August 2020 to July 2021

**DOI:** 10.1038/s41598-022-08684-1

**Published:** 2022-03-18

**Authors:** Wai Sing Chan, Yuk Man Lam, Janet Hei Yin Law, Tsun Leung Chan, Edmond Shiu Kwan Ma, Bone Siu Fai Tang

**Affiliations:** Department of Pathology, Hong Kong Sanatorium and Hospital, Happy Valley, Hong Kong China

**Keywords:** Virology, Viral infection

## Abstract

We extracted one-year genomic data (August 2020–July 2021) from GISAID EpiCoV™ database and estimated monthly proportions of 11 SARS-CoV-2 variants in various geographical regions. From continental perspective, Delta VOC predominated in Africa, Asia, Europe, North America and Oceania, with proportions of 67.58–98.31% in July 2021. In South America, proportion of Delta VOC (23.24%) has been approaching the predominant yet diminishing Gamma VOC (56.86%). We further analyzed monthly data on new COVID-19 cases, new deaths, vaccination status and variant proportions of 6 countries. Delta VOC predominated in all countries except Brazil (Gamma VOC) in July 2021. In most occasions, rise and predominance of Alpha, Beta, Gamma, Delta and Zeta variants were accompanied with surges of new cases, especially after the time point of major lineage interchange. The ascending phases of new cases lasted for 1–5 months with 1.69- to 40.63-fold peak growth, whereas new death tolls varied with regional vaccination status. Our data suggested surges of COVID-19 cases might be predicted from variant surveillance data. Despite vaccine breakthroughs by Delta VOC, death tolls were more stable in countries with better immunization coverage. Another takeaway is the urgent need to improve vaccine efficacy against Delta and emerging variants.

## Introduction

Lives evolve for adaptation and survival, and viruses are no exception. The nucleotide substitution rate of RNA viruses is high, typically ranges from 10^−2^ to 10^−5^ nucleotide substitutions per site, per year (s/n/y)^1^. Severe acute respiratory syndrome coronavirus 2 (SARS-CoV-2), the causative agent of coronavirus disease 2019 (COVID-19), is at the order of 10^−4^ s/n/y^2^. A wealth of mutations has accumulated as the pandemic progressed into second year^3–5^, shaping the genetic diversity of this virus over time, places and hosts.

SARS-CoV-2 is a single-stranded positive-sense RNA virus. Its genome is about 30,000-bp and comprises 11 genes^6^. Among the gene products, spike protein plays a key role in host cell entry, the earliest essential step in viral life cycle^7,8^. This outermost structural protein is thus a prime target of host immune system and under significant selection pressure, as evident by emergence of SARS-CoV-2 variants.

As of 6 July 2021, World Health Organization (WHO) classified common SARS-CoV-2 variants into 4 variants of concern (VOCs), 4 variants of interest (VOIs) and 12 alerts for further monitoring, based on their clinical and public health impact^9^. The 4 VOCs are Alpha (PANGO lineage B.1.1.7), Beta (B.1.351/B.1.351.2/B.1.351.3), Gamma (P.1/P.1.1/P.1.2) and Delta (B.1.617.2/AY.1/AY.2) variants. The 4 VOIs are Eta (B.1.525), Iota (B.1.526), Kappa (B.1.617.1) and Lambda (C.37) variants. The 12 alerts for further monitoring include 3 former VOIs (Epsilon, B.1.427/B.1.429; Zeta, P.2 and Theta, P.3), R.1/R.2, B.1.466.2, B.1.621, AV.1, B.1.1.318, B.1.1.519, AT.1, C.36.3/C.36.3.1 and B.1.214.2.

Continuous surveillance on genomic epidemiology of SARS-CoV-2 is essential to identify variants gifted with growth and transmissibility advantages, so as to keep public health countermeasures and our understanding on the virus up-to-date. The goal of this study was to appreciate recent geographical prevalence of SARS-CoV-2 variants.

## Results

### Prevalence of SARS-CoV-2 variants in 6 continents

**Figure 1:**
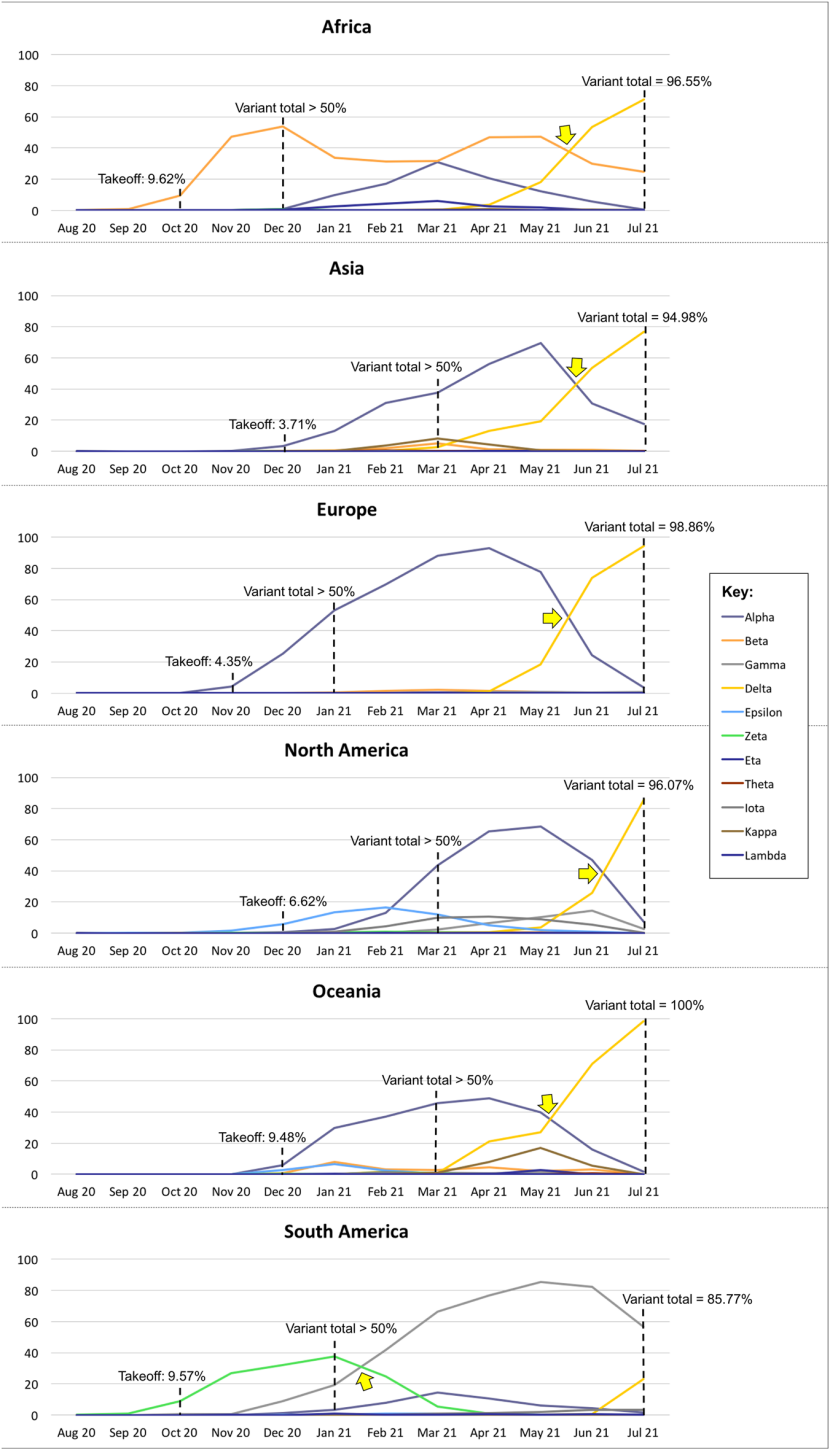
Proportions of SARS-CoV-2 variants collected in 6 continents throughout August 2020 to July 2021. The yellow arrows indicate the time points of major lineage interchange. Please note that owing to highly variable lag time between collection and submission dates, the latest situation in recent months (for instance, June and July 2021) might not be thoroughly reflected.

Results are shown in Fig. 1. Generally, Africa, Europe and South America encountered marked increase of total variant proportion between October and December 2020, and the extent was lesser in Asia, North America and Oceania. The variants expanded and their proportions were over 50% between December 2020 and March 2021, and further rose to 85.77–100% in July 2021.

The 6 continents displayed roughly 4 patterns of variant dynamics. In Africa, proportion of Beta VOC fluctuated over the time and rose by about 5 folds from October 2020 to May 2021. Proportion of Alpha VOC (30.89%) had climbed and approached that of Beta VOC (31.69%) in March 2021, but then declined steadily to 0.41% in July 2021. Delta VOC surpassed Beta VOC in June and reached its prime at 71.27% in July of the same year.

In Asia, Alpha VOC had been the major lineage from December 2020 and rose to the peak of 69.40% in May 2021. It then started to diminish and was replaced by the rapidly expanding Delta VOC in June 2021, with the latter comprising 76.69% of submitted Asian genomes in July 2021. Likewise, Europe and Oceania revealed similar dynamics with higher proportion of Delta VOC in the same month (94.30% and 98.31%, respectively).

In North America, Epsilon variant was the most abundant from late 2020 to early 2021, with relatively low proportions of 5.71–16.42%. It was later overridden by Alpha VOC in March 2021 and the latter being taken over the place by Delta VOC in July 2021.

For South America, Zeta variant outstood other variants from October 2020 (8.89%) to January 2021 (37.51%), followed by Gamma VOC from February 2021 (41.93%) to the peak of 85.29% in May 2021, and dropped to 56.86% in July 2021. Meanwhile, there was more than 20% rise of Delta VOC in the same month.

### Monthly data on new COVID-19 cases, new deaths, vaccination status and variant prevalence of 6 countries

**Figure 2:**
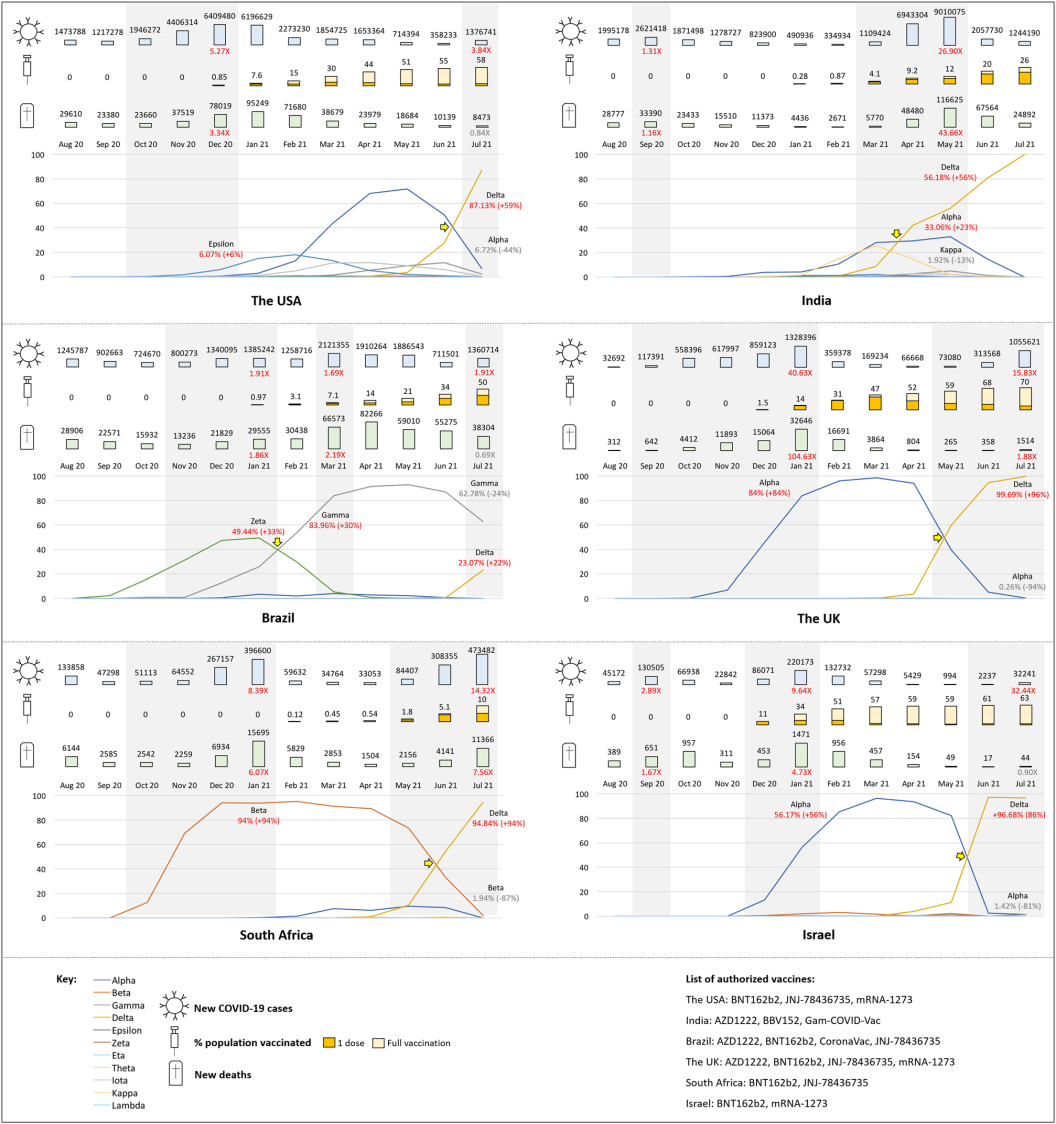
Monthly data on new COVID-19 cases and deaths, vaccination status and proportions of SARS-CoV-2 variants. Grey areas cover the ascending phases of new COVID-19 cases. Yellow arrows indicate the time points of major lineage interchange. The fold and percentage changes were relative to the baseline (the value right before the start of an ascending phase). Please note that owing to highly variable lag time between collection and submission dates, the latest proportions of SARS-CoV-2 variants in recent months (for instance, June and July 2021) might not be thoroughly reflected.

Results are shown in Fig. 2.

### The USA

The USA has been leading in COVID-19 cases and deaths^10^. Her variant dynamics was representative of North America, as 90% of North American genomes were collected in the USA. There were 2 ascending phases of new cases. The first one (October to December 2020) was marked by peak growth of 5.27 folds accompanied with 3.34-fold increase in new deaths, which appeared to be less influenced by the 11 variants due to their low proportions (total variant proportion was 6.89% in December 2020). In between 2 phases, Alpha VOC overrode Epsilon variant in March 2021 and had predominated up till June 2021. Interestingly, new cases and deaths reduced gradually during this period, and the population vaccinated by at least 1 dose rose to 55% (1 dose, 6%; full vaccination, 49%) by June 2021. In the second ascending phase (July 2021), new cases increased sharply by 3.84 folds whereas new deaths decreased by 16.43%, with 58% of population vaccinated (full vaccination: 51%). Notably, it was accompanied with rapid expansion of Delta VOC (+ 59%) and big diminishment of Alpha VOC (− 44%). There were 3 COVID-19 vaccines authorized for emergency use or approved by Food and Drug Administration in the USA, including JNJ-78436735 (Janssen), mRNA-1273 (Moderna) and BNT162b2 (Pfizer-BioNTech)^11^.

### India

India has recorded second highest number of COVID-19 cases in the world, and ranked the third for death toll^10^. There were 2 ascending phases of new cases. The first one was accompanied with short and mild increase of new cases and deaths in September 2020. COVID-19 vaccine was not available at that time, and the proportion of the variants under study was low. The second one (March to May 2021) was marked by large rebound of new cases and deaths (26.9-fold increase from baseline) in an under-vaccinated population (12% by May 2021), involving transition of 3 variants: Kappa VOI diminished from the peak of 25.50% in March to 1.92% in May 2021, with proportions of Alpha and Delta VOCs rising to 33.06% and 56.18% in the same month. In July 2021, Delta VOC expanded to 100% and 26% of Indian population was vaccinated, accompanied with decreasing trends of both new cases and deaths. Three COVID-19 vaccines were approved for emergency use during this period, including BBV152 (Bharat Biotech), AZD1222 (AstraZeneca) and Gam-COVID-Vac (Gamaleya Research Institute of Epidemiology and Microbiology)^12,13^.

### Brazil

Brazil has been ranked the third for COVID-19 cases and the second for death toll^10^. There were 3 ascending phases of new cases. The first one (November 2020 to January 2021) involved about doubled peak growth of new cases and deaths, with 0.97% vaccination coverage by January 2021. Zeta variant comprised the majority (49.44%) and was approached by Gamma VOC (25.81%) in the same month. In the second phase (March 2021), new cases and deaths increased by 1.69 and 2.19 folds, with 7.1% of population vaccinated. The diminishing Zeta variant (5.46%) was superseded by Gamma VOC (83.96%). In the third phase (July 2021), new cases rebounded by about 2 folds and new deaths dropped by 31%, with half of Brazilian population vaccinated. Meanwhile, Gamma VOC has diminished to 62.78% and Delta VOC has expanded to 23.07%. The 4 COVID-19 vaccines deployed for emergency use during this period included CoronaVac (Sinovac Biotech), JNJ-78436735 (Janssen), AZD1222 (AstraZeneca) and BNT162b2 (Pfizer-BioNTech)^14–16^.

### The UK

In the UK, there were 2 ascending phases of new cases. The first one lasted for 5 months (September 2020 to January 2021), with 40.63- and 104.63-fold increase of new cases and deaths in January 2021. Alpha VOC comprised the majority of submitted genomes (83.94%) and 14% of the UK population was vaccinated by the same month. The second one (May to July 2021) was marked by 15.83- and 1.88-fold peak growth of new cases and deaths with 70% vaccination coverage. Delta VOC has superseded Alpha VOC and its proportion has got close to 100% in July 2021. In the UK, 4 COVID-19 vaccines were approved for use, which included mRNA-1273 (Moderna), AZD1222 (AstraZeneca), BNT162b2 (Pfizer-BioNTech) and JNJ-78436735 (Janssen)^17^.

### South Africa

The number of COVID-19 cases in South Africa has been the highest in African continent^10^. There were 2 ascending phases of new cases. The first one lasted from October 2020 to January 2021, with new cases and deaths increased by 8.39 and 6.07 folds, respectively. Vaccination coverage was 0%, and Beta VOC expanded to 93.89% in January 2021. The second one has started from May to July 2021, with 14.32- and 7.56-fold peak growth of new cases and deaths, respectively. By July 2021, Delta VOC (94.84%) has overridden Beta VOC (1.94%), and only 10% of population was vaccinated. In South Africa, BNT162b2 (Pfizer-BioNTech) and JNJ-78436735 (Janssen) vaccines were rolled out for administration^18^.

### Israel

Israel is one of the countries with leading vaccination rate^10^. There were 3 ascending phases of new cases. The first one was in September 2021, with 2.89- and 1.67-fold increase in new cases and deaths. COVID-19 vaccine was not available at that time and total variant proportion was low. The second one (December 2020 to January 2021) was accompanied with 9.64- and 4.73-fold peak growth of new cases and deaths, respectively. By January 2021, Alpha VOC predominated (56.17%) and 34% of Israeli population was vaccinated. The third one (June to July 2021) was marked by a sharp rise of new cases from low baseline (peak growth was 32.44 folds) and 10% drop in new deaths. By July 2021, Delta VOC (variant proportion: 96.68%, increased by 86% from May 2021) surpassed Alpha VOC (variant proportion: 1.42%, decreased by 81% from May 2021) with 63% vaccination coverage. Israel has deployed BNT162b2 (Pfizer-BioNTech) and mRNA-1273 (Moderna) vaccines for administration^19^.

## Discussion

COVID-19 pandemic has been the first of its kind caused by coronavirus. Since its outbreak in December 2019, it has led to 207,784,507 confirmed cases and 4,370,424 deaths worldwide (data accessed on 18 August 2021)^20^. There have been ups and downs of the situation, and in 2021 a number of countries have faced fresh COVID-19 resurgence, including but not limited to the 6 countries in this study. In addition to social factors like public health policy relaxation and pandemic fatigue, emergence of vaccine-escape variants was also an important contributing factor. Our data showed that in July 2021, Delta VOC has become the predominant lineage in Africa, Asia, Europe, North America and Oceania. In South America, its proportion has been increasing (23.24%) and approached that of the predominant yet diminishing Gamma VOC (56.87%), which is not surprising as Delta VOC was reported to be 40–60% more transmissible than Alpha VOC^21^.

From country-level temporal data, we might appreciate the expansion-diminishment patterns of various variants. For instance, the mean expansion-diminishment duration of Alpha VOC was about 7.5 months, and the ascending and descending phases lasted for 4.75 and 2.75 months in average, respectively. In addition, another interesting observation was that the ascending phases of new cases were usually accompanied with rise and predominance of Alpha (except the USA), Beta, Gamma, Zeta, and most recently Delta variants, or right after the time points of major lineage interchange. This might be (or might not be) accompanied with a rise in monthly new deaths, which appeared to be associated with immunization coverage. For instance, proportion of Delta VOC in India rose sharply from March to April 2021, surpassing Alpha VOC and Kappa VOI. In the meantime, both new cases and deaths increased drastically by 26.90 and 43.66 folds in May 2021, and only 8.8% and 3.2% of Indian population received one- and full-dose vaccination, respectively. Similar phenomenon was observed for the under-vaccinated population (in July 2021: one-dose, 5.2%; full vaccination, 4.8%) in South Africa. For the USA, the UK and Israel, more than half of their populations received full-dose vaccination when Delta VOC grew drastically to predominance. Their death tolls remained stable despite surges of new cases. It should be noted that the above observations were based on overall statistical data of each parameter and therefore were approximate. For more detailed analysis on the causal relationships, specific information on, for instance, the vaccination status of infected patients and the deceased, will be needed but we were unable to acquire such data from existing sources. Besides immunization coverage, availability of medical resources might also contribute to difference in mortality^22^.

As of 1 June 2021, WHO validated 5 COVID-19 vaccines for emergency use, including 2 inactivated (Sinopharm and CoronaVac by Sinovac), 2 RNA-based (BNT162b2 by Pfizer-BioNTech and mRNA-1273 by Moderna) and 1 viral vector (AZD1222 by AstraZeneca) vaccines^23^. Emergence of SARS-CoV-2 variants has raised concern over vaccine efficacy against growing variety of lineages. A number of research groups reported vaccine-breakthrough cases involving SARS-CoV-2 variants and nearly all validated vaccines, for instance, Gamma VOC against CoronaVac and AZD1222 in Brazil^24,25^; 2 spike variants harbouring E484K and triple mutations (T95I, del142-144, and D614G) against mRNA-1273 and BNT162b2, respectively in the USA^26^; Alpha and Delta VOCs against AZD1222 and BNT162b2 in England^27,28^ and Scotland^29^; as well as Delta VOC infecting an Everest trekker vaccinated with mRNA-1273 in Nepal^30^. Despite a broad range of cycle threshold values (15–33.3), the authors reported that all these vaccine-breakthrough cases involved reduced disease severity.

On the other hand, a number of studies focused on neutralizing activity of post-second-dose sera. For CoronaVac, Chen and coworkers reported that the neutralizing efficiency against pseudotyped lentiviruses of Beta and Gamma VOCs, and Iota VOI were significantly reduced by factors of 5.27, 3.92 and 4.03, respectively, whereas efficiency against D614G, Alpha VOC and Epsilon variant were equally effective compared with the wildtype^31^. Their data also revealed that 5–34% of sera were capable of neutralizing the former 3 variants, compared to 82% against wildtype. Another study led by Cao and coworkers revealed that Beta VOC pseudovirus or the authentic virus caused a major reduction in neutralization^32^. For BNT162b2, Lustig and coworkers revealed that neutralizing titers against Alpha, Delta and Gamma VOCs were reduced by 1.7–2.6 folds, and 10.4 folds for Beta VOC^33^. For AZD1222, Madhi and coworkers performed both pseudovirus and live virus assays^34^. They observed that 46% and 85% of post-second-dose sera lacked neutralization activity against pseudovirus with triple mutations (K417N, E484K and N501Y) and Beta VOC pseudovirus, respectively. For live virus, 61.54% of post-second-dose sera had undetectable neutralization activity against Beta VOC, and the remainder with detectable activity were reduced by factors of 4.1–31.5. For mRNA-1273, Shen and coworkers reported that neutralization activity against Epsilon variant and Beta VOC pseudoviruses were 2–3 and 9–14 times lower than that against D614G pseudovirus, respectively^35^.

Summing up the serological findings, Delta VOC or other variants reduced neutralizing activity of vaccination-elicited antibodies at varying extents, with Beta VOC being apparently more resistant. Beta VOC is characterized by 9 mutations in S1 subunit (D80A, D215G, 241del, 242del and 243del in N-terminal domain (NTD); K417N, E484K and N501Y in receptor-binding domain (RBD); as well as D614G) and a single mutation in S2 subunit (A701V near S1/S2 furin cleavage site) (Table 1). Among these signature amino acid changes, E484K and combination of K417N, E484K and N501Y might reduce the effectiveness of specific monoclonal antibody treatments^36^. K417, E484 and N501 are 3 of the 21 amino acids in RBD, the major target of neutralizing antibodies^37^, which interact with human angiotensin-converting enzyme 2 (hACE2)^38^. Results of in vitro studies suggested E484K might play an indispensable role in escape of Beta VOC from neutralizing antibodies^39^ and loss of neutralizing activity of certain monoclonal antibodies^39–41^. Despite the difference in resistance against vaccine protection, Beta and Gamma VOCs share very similar RBD mutation pattern, that is, same signature mutations E484K and N501Y, except for K417 which is less impactful than E484 on antibody neutralization. This suggests possible involvement of other mutations, for instance, in NTD, which may contribute to enhanced resistance of Beta VOC against neutralizing antibodies, and this gap of knowledge warrants further investigation.

**Table 1:** Spike protein mutations among SARS-CoV-2 variants of concern (VOCs) and variants of interest (VOIs).

Mutations	VOCs				VOIs							Locations in spike protein	
	Alpha	Beta	Gamma	Delta	Epsilon*	Zeta*	Eta	Theta*	Iota	Kappa	Lambda		
L5F									✓			SP	S1
S13I					✓								
L18F			✓									NTD	
T19R				✓									
T20N			✓										
P26S			✓										
A67V							✓						
69/70del	✓						✓						
V70F				(✓)									
G75V											✓		
T76I											✓		
D80A		✓											
D80G									(✓)				
T95I				✓					✓	(✓)			
D138Y			✓										
141/143del								(✓)					
G142D				✓						✓			
144del	✓						✓		(✓)				
W152C					✓								
E154K										✓			
156/157del				✓									
F157S									(✓)				
R158G				✓									
R190S			✓										
D215G		✓											
A222V				(✓)									
241/242/243del		✓											
243/244del								(✓)					
247/253del											(✓)		
D253G									✓				
W258L				(✓)									
Y265C								(✓)					
K417N		✓		(✓)								RBD	
K417T			✓										
L452Q											✓		
L452R				✓	✓				(✓)	✓			
S477N									(✓)				
T478K				✓									
E484K	(✓)	✓	✓			✓	✓	(✓)	✓				
E484Q										✓			
F490S											✓		
S494P	(✓)												
N501Y	✓	✓	✓					(✓)					
F565L						(✓)							
A570D	✓												
D614G	✓	✓	✓	✓	✓	✓	✓	✓	✓	✓	✓		
H655Y			✓										
Q677H							✓						
P681H	✓							✓					
P681R				✓						✓			
A701V		✓							✓				S2
T716I	✓												
T859N									(✓)		✓		
F888L							✓						
D950N				✓								HR1	
D950H									(✓)				
Q957R									(✓)				
S982A	✓												
T1027I			✓										
Q1071H										✓			
E1092K								✓					
H1101Y								✓					
D1118H	✓												
V1176F						✓		✓				HR2﻿	
K1191N	(✓)												

The cells with ticks and bracketed ticks denote high and partial prevalence in the lineages, respectively. Variants with asterisks are former VOIs. HR1 and 2, heptad repeats 1 and 2; NTD, N-terminal domain; RBD, receptor-binding domain; S1 and S2, subunits 1 and 2; SP, signal peptide.

Among the 6 countries under study, South Africa was the only one which had been predominated by Beta VOC. Proportion of Beta VOC rose from 12.65% in October 2020 to 94% in January 2021, accompanied with the peaks of new COVID-19 cases and new deaths. At that time, COVID-19 vaccination program had not been deployed yet in South Africa. In subsequent 3 months, proportion of Beta VOC was maintained at 89.43–95.45% with significant drop in new cases and new deaths. Meanwhile, vaccination campaign started with less than 1% of South African population receiving one- or full-dose of vaccination, and hence we could not appreciate the impact of Beta VOC to a highly-vaccinated population. For Delta VOC, significant rise in new COVID-19 cases was observed in the USA, India, the UK, South Africa and Israel right after the time point of major lineage interchange, irrespective of vaccination rate.

The unprecedentedly dynamic and vast volume of SARS-CoV-2 genomic data from GISAID database has embodied the power of global and concerted effort on tracking the still-ongoing COVID-19 pandemic. The database is very informative and user-friendly, however, care must be taken when drawing conclusions based on the sequence data, and the following considerations infer the limitations of this study. First, as individual sequences were processed and submitted by various institutes worldwide, there might be variation in quality that we could hardly monitor. For instance, details on coverage and assembly method of some sequences were not provided by the submitters. Second, our data might not reflect the latest situation (especially for June and July 2021) due to highly variable lag time between collection and submission dates. Another factor to consider was inhomogeneous sampling. For instance, at continent level, sequences submitted by the USA comprised about 90% of North American sequences, and the situation of other countries might be under-represented. At country level, we could not ensure submitted sequences were representative of the population without sampling bias towards particular lineages, nor for statistical data such as reported death tolls in certain countries. Another limitation was that we could not distinguish between locally transmitted and imported COVID-19 cases.

To conclude, our data showed that up till July 2021, Delta VOC has predominated in 5 continents, and it appeared to be likely to replace Gamma VOC in South America in near future. By monitoring prevalence of SARS-CoV-2 variants, we can track the emergence of ‘the fittest and the fastest’, and combination of variant prevalence and public health surveillance data might be useful for predicting next surge of COVID-19 cases. Surveillance of SARS-CoV-2 variants is particularly important for under-vaccinated regions with limited medical resources to minimize fatality. The breakthrough by Delta VOCs in countries with higher vaccination rate suggested a broader immunization coverage to reach the threshold of herd immunity to reduce overloading of medical systems and vaccines with improved efficacy against major variants.

## Methods

### SARS-CoV-2 variants

This study focused on 4 VOCs (Alpha, Beta, Gamma and Delta), 4 VOIs (Eta, Iota, Kappa and Lambda) and 3 former VOIs (Epsilon, Zeta and Theta).

### Geographical prevalence of SARS-CoV-2 variants

Geographical prevalence of selected variants was estimated from monthly proportions of whole genome sequences submitted to Global Initiative on Sharing All Influenza Data (GISAID) EpiCoV™ database^42^. Data was extracted with following search criteria: complete genome (> 29,000 bp), high coverage (< 1% undefined bases), human host, collection date in 12 separate months (August 2020 to July 2021), location covering 6 continents (Africa, Asia, Europe, North America, Oceania and South America) and submission date up to 7 July 2021 for August 2020 to June 2021, and up to 8 August 2021 for July 2021. Please note that owing to highly variable lag time between collection and submission dates, the latest situation in recent months (for instance, June and July 2021) might not be thoroughly reflected.

### Further study on countries under the spotlight

These included the USA, India, Brazil, the UK, South Africa and Israel. We looked for potential relationship between monthly data on variant proportions, new COVID-19 cases and deaths, and vaccination status in these countries. Variant prevalence data was extracted from GISAID EpiCoV™ database as described above, and figures on new COVID-19 cases and deaths, and percentage of population vaccinated with 1 and full dose were extracted from Google News COVID-19 page^10^.

### Ethics approval and consent to participate

Not applicable. All human-related statistical data (number of new COVID-19 cases, vaccination status and number of new deaths) were adopted from Google News (cited as reference^10^).

### Software for image creation

Both Fig. 1 and 2 were created by the authors using Microsoft Office 365.

## Data Availability

The datasets used and/or analyzed during the current study are available from the corresponding author on reasonable request.
